# Effectiveness of Psychoeducational Group Training on Quality of Life and Recurrence of Patients with Bipolar Disorder

**Published:** 2017-01

**Authors:** Farhad Faridhosseini, Mehdi Baniasadi, Mohammad Reza Fayyazi Bordbar, Meysam Pourgholami, Samira Ahrari, Negar Asgharipour

**Affiliations:** 1 Associate Professor of Psychiatry, Psychiatry and Behavioral Sciences Research Center, Ibn-e-Sina Hospital, Faculty of Medicine, Mashhad University of Medical Sciences, Mashhad, Iran.; 2 Psychiatrist, Psychiatry and Behavioral Sciences Research Center, Ibn-e-Sina Hospital, Mashhad, Iran.; 3 Professor of Psychiatry, Psychiatry and Behavioral Sciences Research Center, Ibn-e-Sina Hospital, Faculty of Medicine, Mashhad University of Medical Sciences, Mashhad, Iran.; 4 Psychiatrist and Fellow in Psychotherapy, Mental Health Research Center, Tehran Institute of Psychiatry, School of Behavioral Sciences and Mental Health, Iran University of Medical Sciences, Tehran, Iran.; 5 Assistant Professor of Clinical Psychology, Psychiatry and Behavioral Sciences Research Center, Ibn-e-Sina Hospital, Faculty of Medicine, Mashhad University of Medical Sciences, Mashhad, Iran.

**Keywords:** *Bipolar Disorder*, *Group Training*, *Psychoeducation*, *Quality of Life*, *Recurrence*

## Abstract

**Objective: **Bipolar disorder (BD) is a disabling psychiatric disorder with frequent recurrences. Besides pharmacotherapy, psychoeducation could be helpful in reducing symptoms as well as recurrence of this disorder, leading to improvement of patients’ quality of life. This study aimed at investigating the effectiveness of a culturally adjusted structured program for training Iranian BD patients.

**Method:** In a 6-month course (spring and summer 2014), 24 BD patients, visiting the outpatient clinic of Ibn-Sina Hospital in Mashhad and experiencing euthymic phase, were allocated in to 2 groups of intervention and control. The intervention group received 8 sessions of psychoeducation in four weeks. Patients in the control group received the usual treatment. The patients were evaluated with Hamilton Depression Rating Scale, Young Mania Rating Scale, and Short Form 36 before the intervention and 4 weeks later, and the results were compared using independent t test. The patients were reexamined after 6 months for recurrence, hospitalization, treatment adherence, and visiting a psychiatrist, and were compared with patients in the control groups.

**Results:** There was a significant difference in the intervention group in improvement in quality of life before and after treatment (p<0.003). In addition, the difference was significant between the 2 groups in the number of recurrence (p<0.001) and hospitalization (p<0.000) in 6 months.

**Conclusion**: In addition to pharmacotherapy, psychoeducation of patients with BD can improve their quality of life and decrease the risk of disorder recurrence.

According to the World Health Organization (WHO), mood disorders are important health issues of the 21st century (1). Multiple studies on bipolar disorder show that it has taken the sixth or seventh rank among other debilitating disorders worldwide (2, 3). The disorder will incur huge costs to the patients and the societies (4). Recent studies by the World Federation for Mental Health conducted in 11 countries, have reported the Prevalence rate of BD as 2.4%, but only less than half of the patients with BD receive treatment (5). 

In low-income countries, only 25% of the patients with BD were in contact with the mental health system.

Despite little request from BD patients for receiving the required treatment, considerable advancements have been made in curing this problem (6).

Although pharmacotherapy is essential for successful treatment, it cannot completely prevent the recurrence of the disorder. Nearly 40%, 60%, and 75% of the BD patients, who are under pharmacotherapy, have recurrence in the first, second, and third year, respectively.

In addition, a considerable portion of the patients (more than 50% in some researches) experience interepisode residual symptoms (7-9).

Due to the chronic nature of BD, patients will face problems in occupational and interpersonal relationships, and the rate of divorce has been reported very high among this group of patients (10). In spite of patients’ improvement during the active phase of the disorder, their quality of life remains impaired and the rate of suicide has been reported very high among this group of patients as well (11). 

Several studies have highlighted the role of training in the treatment of bipolar disorder, and it seems helpful in reducing the recurrence, burden, and progress of the disorder, as well as improving the patient’s functioning. Polak suggests that training the patients should begin after their discharge from hospital, with more emphasis on enabling them to evaluate their symptoms and acquire some skills in this regard (12, 13). However, planning interventions regarding specific needs of patients before discharge could improve the outcome (14). A main reason for recurrence of BD is non-compliance by the patients (15, 16). This can be due to the lack of knowledge about drug side effects, the need for continuing the treatment, and fear of addiction to medication etc. (17).

The contents of training programs focus mainly on the knowledge of the patients about the disorder, triggers of new episodes, the need for treatment, different therapeutic options, coping with mood fluctuations, achieving higher control over the symptoms, understanding medication side effects, and the significance of regular habits and relatively organized life style (18- 21). Iker and Harkin investigated the effectiveness of a 6-week training program on treatment adherence of BD patients. Their results showed that the rate of treatment adherence increased from 40% in the pretest to 86% in the posttest (22). Despite extended studies on this area over the past 3 decades, the need for empowering and increasing such research, especially in the field of designing and evaluating indigenous intervention strategies, is felt (23). 

Studies conducted in Iran on the effectiveness of psychoeducation-based interventions have focused on the training the patients’ families (24- 26). To our best knowledge, no study has been conducted on the effectiveness of a comprehensive therapeutic psychoeducational package for patients. This study investigated the quality of life, the level of depression, and changes in the Young Mania Rating Scale in patients receiving 8 weeks of group psychoeducational training program. In addition, the effect of interventions on the variables, the rate of recurrence after 6 months, treatment compliance, hospitalization, and the number of psychiatric visits were compared between the 2 groups.

## Materials and Methods

The research study utilized clinical trial design. Based on the study by Iker and Harkin’s, the sample size of 13 was selected for each group. Sampling method was convenience purposive sampling, and significance level was set at 95% (α = 0.05) and a test power of 80% (β = 0.02). The inclusion and exclusion criteria were given to the psychiatrists based on which they referred eligible patients to the therapist for initial assessment. The inclusion criteria were as follow: patients with BD diagnosed based on DSM-IV-TR criteria, being in euthymic phase of disorder, having at least a junior high school degree, aged 18 to 50 years, no history of psychotherapy or other major psychiatric disorders, and completed written consent form (the patients had the capacity to be informed about the purpose of the study). Patients were excluded from the study in case of dissatisfaction of treatment or recurrence of the disorder during the training sessions. The patients whose recurrence occurred after the psychoeducational course were not excluded; they completed the 6- month of follow- up period and were assessed for recurrence and hospitalization. The therapist selected euthymic patients based on a detailed clinical interview, and obtained their written consent. Patients who agreed to participate in group therapy were included in the process of assessment and treatment. Then, the patients were randomly allocated to the psychoeducation and control groups based on the random number table. To prevent patients from leaving the study, sessions were held twice per week for one month in the Ibn-Sina Hospital of Mashhad during spring and summer 2014. The patients were evaluated with Hamilton Depression Rating Scale, Young Mania Rating Scale, and Short Form Health Survey (SF36) before and after the intervention. The patients were reexamined after 6 months. The number of recurrence, hospitalization, medical compliance, and being visited by the prescribing psychiatrist were compared between the 2 groups. The rate of compliance was defined based on their regular use of medication. The patients’ anonymity was preserved. The patients in the control group were also assessed with Hamilton Depression Rating Scale, Young Mania Rating Scale, and Short Form 36 before and after the intervention. A local need- based psychoeducational program designed by Tabatabaei and Mottaghipour was used. The preparation phases of the program had been described previously (19). The general contents of the sessions are as follow:

- The first session: the concept of bipolar disorder and its etiology
- The second session: mania and hypomania
- The third session: depression and mixed episodes
- The fourth session: clinical course and prognosis
- The fifth session: knowing the mood stabilizers and their side effects
- The sixth session: knowing antidepressant and antimanic medications and their side effects
- The seventh session: early detection of new episodes
- The eighth session: what to do when a new episode is detected

The inclusion criteria were as follow: patients with BD diagnosed based on DSM-IV-TR criteria, being in euthymic phase of disorder, having at least a junior high school degree, aged 18 to 50 years, no history of psychotherapy or other major psychiatric disorders, and completed consent form. Patients were excluded from the study in case of dissatisfaction with the treatment or recurrence of the disorder during the training sessions. The patients whose recurrence occurred after the psychoeducational course were not excluded; they completed the 6 month follow up period and were assessed for recurrence and hospitalization.

***The Study Instruments***

**1.           Hamilton Depression Rating Scale (HDRS):** As a clinical trial scale for assessment of depression, it has 17 items and evaluates behavioral, physical, and mental representation of depression. This questionnaire was translated into Persian by Mahyar and Mousavi Nasab in 1986. In some studies, score of 16 has been considered as the cut-off point for this scale. In previous studies by Hamilton, using correlation coefficient, inter-ratter reliability of 0.90 to 0.094 was reported for the questionnaire. Validity of the scale ranged from 0.60 to 0.84 based on the correlation with other instruments. In addition, its internal validity was 0.84 to 0.90. Gharae, Mahyar, and Mehrabi (2000) reported the reliability of the scale as 0.85 and 0.89, respectively, using test-retest analysis. Tozendehjani and Abdollahian reported the correlation coefficient of the Beck Depression Inventory and HDRS as 0.65 (4). 

**2.           Young Mania Rating Scale (YMRS):** This 11-item scale is used to objectively score the severity of BD during the manic episode. YMRS total scores range from 0 to 60. It is translated into Persian, showing acceptable reliability and validity based on a study among Iranian patients in Isfahan (27). Also, it is validated in several studies in other countries (28- 30).

**3.           Short Form 36:** It is a general scale for assessing quality of life. It is a 36-item self-reporting instrument assessing 8 subscales, namely, physical performance, physical roles, physical pain, general health, vitality, social performance, emotional performance, and mental health. These 8 subscales can be evaluated from physical and mental perspectives. Each SF-36 subscale scores from 0 to 100. The more the scale is closer to 100, the higher is the quality of life. The validity and reliability of the Persian version of SF-36 have been examined in Iran, suggesting the score suitable for assessment of health related quality of life (31). Internal consistency analysis revealed that the Persian version of SF-36 had minimum standard reliability of 0.77 to 0.90. All correlation coefficients were greater than 0.40 (ranging from 0.58 to 0.95). Moreover, Miler et al. confirmed the validity and reliability of the SF-36 test in 2016 (32).

***Statistical Analysis***

 Distribution of quantitative variables was analyzed with Kolmogorov-Smirnov test. The paired t test was used to compare quantitative variables between the two groups with normal distribution including depression score and SF36. In addition, Wilcoxon test was used to compare the quantitative variable with skewness including Young’s mania score. The investigated variables in the treatment and control groups were studied, and the degree of changes was calculated before the study and 4 weeks after the completion of the study. After 6 months, the patients in both groups were assessed for recurrence of the problem, hospitalization, visiting psychiatrist, and compliance with chi-square test.

## Results

Thirteen patients in the treatment group received psychoeducation. In the second session, one patient was excluded from the study due to the recurrence of manic symptoms and referred to a psychiatrist. In addition, a patient in the control group did not show up for the final assessment because of unwillingness to cooperate. The intervention group consisted of 6 males and 6 females with the mean age of 28.58±6.52 years; the control group consisted of 7 males and 5 females with the mean age of 28.25±6.55 years. The average number of episodes in the intervention and control groups was reported to be 4.91±4.01 and 4.50±0.90, respectively. The mean number of hospitalization was 2.79±2.00 and 1.58±1.50 in the intervention and control groups, respectively. The duration of the disorder was reported to be 6.25±4.95 and 7.25±4.20 years in the intervention and control groups, respectively. No significant differences were detected in the demographic data of the intervention and control groups (p>0.05). Results are presented in Table 1.

Comparison of pretest vs. posttest scores regarding the level of depression was done by HDRS, score of YMRS, and quality of life of SF-36, which is presented in Table 2. In contrast to the control group, a significant difference was observed in the intervention group in the SF-36 score. An insignificant difference was observed in this group in depression score. 

Based on the results of the independent t test, differences between the 2 groups was addressed in the posttest. The results are presented in Table 3. A significant difference was observed in the scores of YMRS in the fourth week of the study in the intervention group.

The patients were re-examined 6 months after the completion of the study.

They were evaluated for the number of recurrence, hospitalization in a psychiatric hospital, degree of compliance, and number of being visited by their psychiatrist.

In the intervention group, recurrence occurred only once in one patient, while in the control group it occurred 9 times in 7 patients.

Two patients in the control group had 2 recurrences in 6 months. None of the patients in the intervention group was hospitalized, while 4 were hospitalized in the control group.

All the patients in the intervention group had medicinal compliance based on their self- report as well as their caregivers’ report.

**Table1:** Comparison between Two Groups in Demographic Information

**Groups**	**Number of Patients**		**Age**		**Number of Episodes**		**Number of Hospitalization**	
	**Female**	**Male**	**Mean**	**SD**	**Mean**	**SD**	**Mean**	**SD**
Intervention	6	6	28.58	6.52	4.91	4.01	2.00	2.79
Control	5	7	28.25	6.55	4.50	0.90	1.50	1.58

*p*>0.05

**Table2:** Comparison of Pretest vs. Posttest Scores of HDRS, YMRS, and SF-36 (Paired t Test for HDRS & SF-36; Wilcoxon Test for YMRS)

**Indices** **Group**	**HDRS**				**YMRS**				**SF-36**			
	**Mean±SD**		**Mean change**	**p-value**	**Mean±SD**		**Mean change**	**p-value**	**Mean±SD**		**Mean change**	**p-value**
	**Week 0**	**Week 4**			**Week 0**	**Week 4**			**Week 0**	**Week 4**		
intervention	10.75 8.22±	8.25 5.59±	2.50 ±4.52	0.082	2.08 2.10±	0.08 0.28±	2.38	0.17	70.00 15.77±	75.29 16.94±	5.28 ±4.70	0.003
control	7.33 4.18±	5.83 3.32±	1.50 ±4.23	0.245	2.25 1.42±	2.16 1.69±	0.775	0.439	71.81 13.53±	73.97 15.32	2.1 5±6.62	0.283

**Table3:** Comparison between the Indices of the Investigated Variables in Two Groups in the Fourth Week Based on the Independent t Test

**Group**	**HDRS**			**YMRS**			**SF-36**		
	**Mean**	**Standard ** **Error**	**p-value**	**Mean**	**Standard ** **Error**	**p-value**	**Mean**	**Standard ** **Error**	**p-value**
Intervention/Control	1.00	1.78	0.582	1.91	0.88	0.041	3.12	2.34	0.196

**Table4:** Comparison between Two Groups in the Number of Recurrence, Hospitalization, and Visiting a Psychiatrist Six Months after the Completion of the Study Based on the Independent t Test

**Group**	**Evaluation of the Number of ** **Recurrence**			**Number of Hospitalization**			**Number of Visiting a ** **Psychiatrist**		
	**Mean**	**SD**	**p-value**	**Mean**	**SD**	**p-value**	**Mean**	**SD**	**p-value**
Intervention	0.083	0.288	0.001	0.000	0.000	0.000	3.25	0.965	0.021
Control	0.750	0.753		0.333	0.492		1.41	1.67	

However, in the control group, 6 patients had regular drug consumption and 6 had irregular drug use or stopped using it. The questions regarding medicinal compliance were asked from the patients and their families during the interview, and the answers were then entered into the questionnaire. Results of the comparison between the t2 groups are presented in Table 4. A significant difference was observed between the 2 groups in all the cases.

## Discussion

In the present study, the effects of group psychoeducation were evaluated on BD patients. The efficacy of an 8-session training package, designed by Tabatabaei and Mottaghipour, on the BD patients was investigated for the first time in Iran. As a result, an improvement in the quality of life of the intervention group was observed in SF-36 scale. In addition, the score of YMRS reduced in this group after 4 weeks of intervention. During the 6 months follow- up, the number of recurrence and hospitalization decreased significantly in the intervention group compared to the control group; treatment adherence was also clearly increased in the intervention group.

Most researches conducted on the psychoeducation in bipolar disorder have shown that it can improve the course of disorder both individually or as a part of a general program. Providing the patients and their families with information about the disorder and therapeutic methods could decrease the recurrence of manic or depressive episodes and the need for hospitalization. It also increases the inter-episodic intervals and acceptance of the treatment, as well as decreases symptoms (33). Another study in Japan found that a simple psychoeducational program could improve the mood recognition in patients with bipolar II disorder (34).

 Colom et al. emphasized that psychoeducation should be taken into consideration more than any other psychotherapeutic modality as a part of the disorder management program (35). In addition, they argued that training to encourage discipline in everyday life could play an essential role in preventing depression. Moreover, recognizing prodromal symptoms of the episode could play a significant role in preventing mania (36). Group psychoeducation for a course of 6 months could bring long-term preventive impacts in euthymic bipolar patients in a 5-year follow- up (37). In the present study, conducted for the first time in Iran, the course of therapy lasted for the shortest possible period (4 weeks) due to the low acceptance by the patients. This indicates that psychoeducation is associated with obvious improvement in the quality of life of the patients even in a short-term course. On the other hand, psychoeducation has its maximum influence when the patient is in the euthymic phase (35). 

In previous studies, a salient difference has been observed in the number of recurrence between those patients who received psychotherapy and the members of the control group. In a 5-year follow- up, Colom confirmed that the number of BD episodes in the psychoeducation group was much less, and manic prevention was much more successful in a long-term period. This may be due to more discipline in life habits and especially early detection of the symptoms in the long-term (38). In addition, Colom states that the time spent with subsyndromal symptoms significantly decreased in the psychoeducation group, so these patients spent up almost 8% of the study time ill, while this rate was up to 30% in other patients. They spent 66% less time in mania, hypomania, and mixed phases, and 75% less time in depression (38). Again, psychoeducation seems to have more effect on duration of an acute condition than other nonpharmacologic treatments including behavior therapy (39). In the present study, the 6 months follow- up revealed a significant difference in the number of recurrence between the 2 groups, indicating the effect of group psychoeducation in reducing the number of recurrence.

The effect of psychoeducation on the frequency of hospitalization is also highly remarkable. In the 6-month follow-up of the present study, the number of hospitalization in the psychoeducation group significantly decreased compared to the control group. 

One hypothesis for the efficacy of psychoeducation on course of bipolar disorder is that the training could lead patients to be more capable of detecting the prodromal symptoms and contacting the psychiatrist before the disorder severity necessitates hospitalization. In a similar study, no significant difference was observed in the number of patients in need of hospitalization in 2-year and five-year follow-ups, but the number of hospitalization decreased for each individual, meaning that psychoeducation could significantly prevent frequent hospitalization of the patients. This effect was not observed in BD patients treated with other psychotherapeutic modalities (38). In addition, Simon et al. reported that although a systematic care program for bipolar disorder can significantly decrease the number and severity of manic episodes, it does not affect the frequency of hospitalization (40). 

One of the main objectives of psychotherapy is increasing treatment adherence (41). Results published by Mosley revealed that fear of becoming dependent is the main reason for the lack of treatment adherence by the patients (42). Colom and Vieta stated that to increase patients’ medicinal compliance, other psychotherapeutic interventions should be employed in addition to simply informing patients (43). On the other hand, it has been mentioned in some studies that psychotherapy has insignificant effects on the patients’ adherence to treatment because of lack of knowledge unless some psychoeducational training programs have been added to the course. Moreover, an assumption maintains that treatment adherence decreases over time, highlighting the need for booster sessions (38). In the present study, a 6-month follow-up was conducted and revealed that therapeutic adherence was significantly different in the psychotherapy group relative to the control group. However, adherence was assessed on patients and their family’s self-report, and we did not assess medication adherence. We did not match the 2 groups for medication adherence, however, both groups were samples among the patients with recurring course of illness and history of multiple hospitalizations. Hence, it is probable that the difference among the groups was due to the difference in their compliance at the beginning of the follow- up.

Psychoeducation as a form of psychotherapy could be useful in the long- term management of patients; however, clinicians should be aware of some side effects as every other treatment. Investigating the negative effects of psychotherapy is essential for bipolar patients and could be proposed as the subject of future research. In a review of studies about psychoeducation and cognitive-behavioral therapy in BD patients, Gonzales et al. discussed the probable increased consumption of antidepressants and developing depression during psychotherapy (44). Vieta emphasized that psychotherapy may not be suitable for every BD patient. For example, in patients with obsessive disorder such treatment may lead to the increased number of unplanned psychiatrist visits and drug overdose. Some of those patients may apply strict rules to themselves and their life related issues. For example, they may enforce rigid rules to their sleep pattern, social interaction, and their activity schedule and follow them strictly (45). Vieta also states that those patients who still have symptoms and experience frequent episodes are probably prone to distress and sometimes exacerbation due to psychological interventions (1, 25). It seems that patients with depression are more likely to absorb negative aspects of psychotherapy, and the patients with mania may never apprehend the concepts over psychotherapy (46). In a study by Colom, 3 BD patients under psychotherapy reported increased fear of depression and isolation. In that study, one patient obsessively began to check his mood and reported his daily mood swings the over 3 weeks after the sessions. Although most of the mentioned changes were transient, the idea that psychotherapy is free of negative impacts is unrealistic (38). In a study by Scott et al., patients with more than 12 episodes did not respond well to an intensive course of cognitive-behavioral therapy (47). Assessment of other variables that are related to weak prognosis is very important for predicting a poor response to psychotherapy in the future researches, leading to better modification of combination treatment to enhance prognosis.

Few studies have compared the effects of different psychotherapeutic methods. Most of these researches focus on the early recognition of the signs of treatment adherence, stability of sleep-wake cycle, cognitive reconstruction, and family relations (48). 

Further investigations are required to clarify the effect of each psychotherapy model on different disorder aspects such as depression and mania (48). In addition, the most appropriate psychotherapy method for each specific population of BD patients, like those with long history of recurrence, bipolar II disorder, rapid cycling BD, cyclothymic BD, and other cases with poor prognosis should be recognized (49). On the other hand, the ideal number of psychotherapy sessions for BD patients has not been answered yet. In previous investigations, participation in 13 to 15 sessions led to stable findings regarding the treatment outcome (50). The ideal number of treatment sessions for each patient depends on the onset of the last episode, disorder complexity, associated problems, social support, and response to t treatment (48). Specification of the ideal treatment period requires huge number of studies in which the effects of variety of therapeutic methods with different duration and frequency are investigated on patients with different types of bipolar disorder (51).

## Conclusion

In general, psychotherapy should be taken into consideration as a method for prevention of new episodes (38). The available therapeutic instructions recommend the application of psychotherapy for curing bipolar disorders and propose such interventions, like psychotherapy, as an option for treating BD (35).

## Limitations

Regarding limitations, since the minimum level of educational as an inclusion criterion was high school diploma and the age range of 18 to 50 years was considered, a number of patients in euthymic phases were excluded in spite of having other criteria, leading to small sample size. Therefore, our results could not be generalized to all patients with bipolar I disorder. Following- up the patients for a period of 6 months could be considered as the strength of the study, however, we did not assess the residual mood symptoms during the follow- up. Moreover, we evaluated and compared the adherence of patients just by their family and self-report. Therefore, it could not be ruled out that the difference among the 2 groups in recurrence rate may be due to the differences in the compliance before our intervention.
